# Intraoral Neuromuscular Stimulation Device and Rapid Eye Movement-Dependent Obstructive Sleep Apnea

**DOI:** 10.7759/cureus.27418

**Published:** 2022-07-28

**Authors:** Waiz Wasey, Naila Manahil, Neha Wasey, Sharefi Saleh, Asiya Mohammed

**Affiliations:** 1 Family and Community Medicine, Southern Illinois University School of Medicine, Springfield, USA; 2 General Practice, Shadan Institute of Medical Sciences, Hyderabad, IND; 3 Family Medicine, Ruth Temple Clinic, Los Angeles, USA; 4 Family Medicine, Southern Illinois University Center for Family and Community Medicine, Springfield, USA

**Keywords:** muscle skeletal, rem, neuromuscular electrical stimulation, motor neurons, obstructive sleep apnea (osa)

## Abstract

Obstructive sleep apnea (OSA) is a sleep breathing disorder characterized by recurrent pharyngeal collapse secondary to the decreased tone of the pharyngeal dilator muscles. The genioglossus muscle is a major pharyngeal dilator responsible for maintaining the upper airway. Research has shown that patients with OSA have a stronger but less endurant genioglossus muscle. Research has also demonstrated that neuromuscular electrical stimulation of the skeletal muscles in the genioglossus was associated with improvement in muscular endurance and hence improvement in mild OSA. This has led to the development of a novel intraoral neuromuscular stimulation device for treating snoring and mild OSA. It is known that OSA is worse in rapid eye movement (REM) sleep compared to other stages of sleep due to neurologically mediated impairment of skeletal muscles. What has not been demonstrated so far is if the intraoral neuromuscular stimulation device improves the apnea-hypopnea index (AHI) in REM sleep. Our case report highlights the significant improvement of REM-dependent OSA in a middle-aged female with consistent use of an intraoral neuromuscular stimulation device marketed as eXciteOSA® (Signifier Medical Technologies, Needham, MA).

## Introduction

Obstructive sleep apnea (OSA) is a sleep-related breathing disorder characterized by a decrease or complete cessation of airflow despite an effort to breathe [1]. The cessation of airflow is secondary to recurrent pharyngeal collapse as a result of decreased tone of the pharyngeal dilator muscles [2]. The genioglossus muscle is a major pharyngeal dilator responsible for maintaining the upper airway. Various studies have shown that the genioglossus muscle demonstrates less endurance in patients with OSA [3].

Research has demonstrated that neuromuscular electrical stimulation of the skeletal muscles was associated with improvement in muscular endurance. Both type 1 and type 2 skeletal fibers showed significant muscle hypertrophy [4]. A recent study showed that daytime neuromuscular electrical training of the tongue muscles, especially the genioglossus, was effective in reducing objective and subjective snoring [5]. It also led to a reduction in daytime sleepiness and improved sleep quality. Consistent use of the intraoral stimulation device has been reported to improve apnea-hypopnea index (AHI) by at least 50%. What has not been studied or documented is the effect of these devices on rapid eye movement (REM) sleep-associated AHI and OSA.

It is well established that OSA is worse during REM sleep as a result of neurologically mediated impairment of skeletal muscles. We report the first documented case of significant improvement in REM AHI using a novel intraoral neuromuscular stimulation device.

## Case presentation

A 47-year-old female was seen in the sleep clinic for snoring, frequent nocturnal awakenings, and nonrestorative sleep. Her medical history included bipolar disorder, hypothyroidism, and hyperlipidemia. She was not taking any opioids or central nervous system depressants. She had an initial home sleep study in 2015, which showed mild OSA with an AHI of 5/hour. She was not treated for this. At our consultation visit, she had an Epworth score of 10 and reported worsening of her snoring. A home sleep study using WatchPAT (Itamar Medical, Caesarea, Israel) was ordered. The results of this study were as shown in Table 1.

**Table 1:** Home sleep study, February 2021 REM: rapid eye movement; AHI: apnea-hypopnea index.

Total sleep time (TST)	4 hours 4 minutes
REM sleep (%)	22.9%
AHI	10.6/hour
AHI in REM sleep	41.7/hour

We discussed treatment options with the patient. Although overall her sleep apnea was mild, but since her AHI in REM sleep was in the severe range and her Epworth score was 10, we recommended continuous positive airway pressure (CPAP) device therapy. She was initiated on auto CPAP with a range of 6-15 cmH2O pressure. Over the next four months, the patient struggled with claustrophobia, mask fitting, and compliance issues. Appropriate steps were taken for each of the difficulties the patient was facing, but at the end of four months, the patient refused to invest more effort in CPAP treatments and wanted to explore other options.

Position therapy was not an option as there was no significant AHI difference with the position. A mandibular advancement device was not recommended by the patient's dentist due to moderate temporomandibular joint (TMJ) disorder. She was not a candidate for a hypoglossal nerve stimulation implant (Inspire) either. Finally, we discussed eXciteOSA® (Signifier Medical Technologies, Needham, MA). The 20-minute daily daytime therapy piqued the patient's interest and she believed that she could be more compliant.

The therapy with neuromuscular electrical stimulation was started and continued for six weeks. The patient reported no difficulties, pain, or compliance issues. She reported less snoring, more refreshed sleep, and decreased nocturnal awakenings. A repeat home sleep study was done with the same WatchPAT device at the end of six weeks of treatment. The results are illustrated in Table 2.

**Table 2:** Home sleep study, November 2021 REM: rapid eye movement; AHI: apnea-hypopnea index.

Total sleep time (TST)	4 hours 3 minutes
REM sleep (%)	25.1%
AHI	0.5/hour
AHI in REM sleep	0.0/hour

The repeat study showed resolution of her OSA, and surprisingly also of the severe OSA in REM sleep. Epworth's score improved to 2, and she continued with the maintenance therapy sessions twice a week. She again had another polysomnography in June 2022 that showed the following results (Table 3).

**Table 3:** Polysomnography, June 2022 REM: rapid eye movement; AHI: apnea-hypopnea index.

Total sleep time (TST)	6 hours 16 minutes
REM sleep (%)	15%
AHI	0.3/hour
AHI in REM sleep	2.1/hour

## Discussion

OSA is a breathing disorder characterized by the recurrent pharyngeal collapse of the upper airway leading to a decrease or a complete cessation of airflow [1]. These events occur despite an effort to breathe. The decreased tone of the pharyngeal dilator muscles, especially the genioglossus, is primarily responsible for recurrent OSA events. Studies have demonstrated that the genioglossus muscles are stronger but less endurable in patients with OSA [3].

Neuromuscular electrical stimulation studies on skeletal muscles have demonstrated that repeated stimulation targeted to these muscles was associated with improvement in endurance [4]. Muscle biopsy indicated that both type 1 and type 2 skeletal muscles showed significant hypertrophy with neuromuscular electrical stimulation. This led to experiments into stimulating the genioglossus muscles with the aim of improving endurance and hopefully airway patency in patients with OSA. A recent publication by Baptista et al. (2021) studied the effects of daytime neuromuscular electrical training on the tongue muscles. It was noted that the repeated daytime stimulation led to improvement in both objective and subjective symptoms of snoring. Furthermore, improvements were noted in daytime sleepiness and sleep quality.

**Figure 1:**
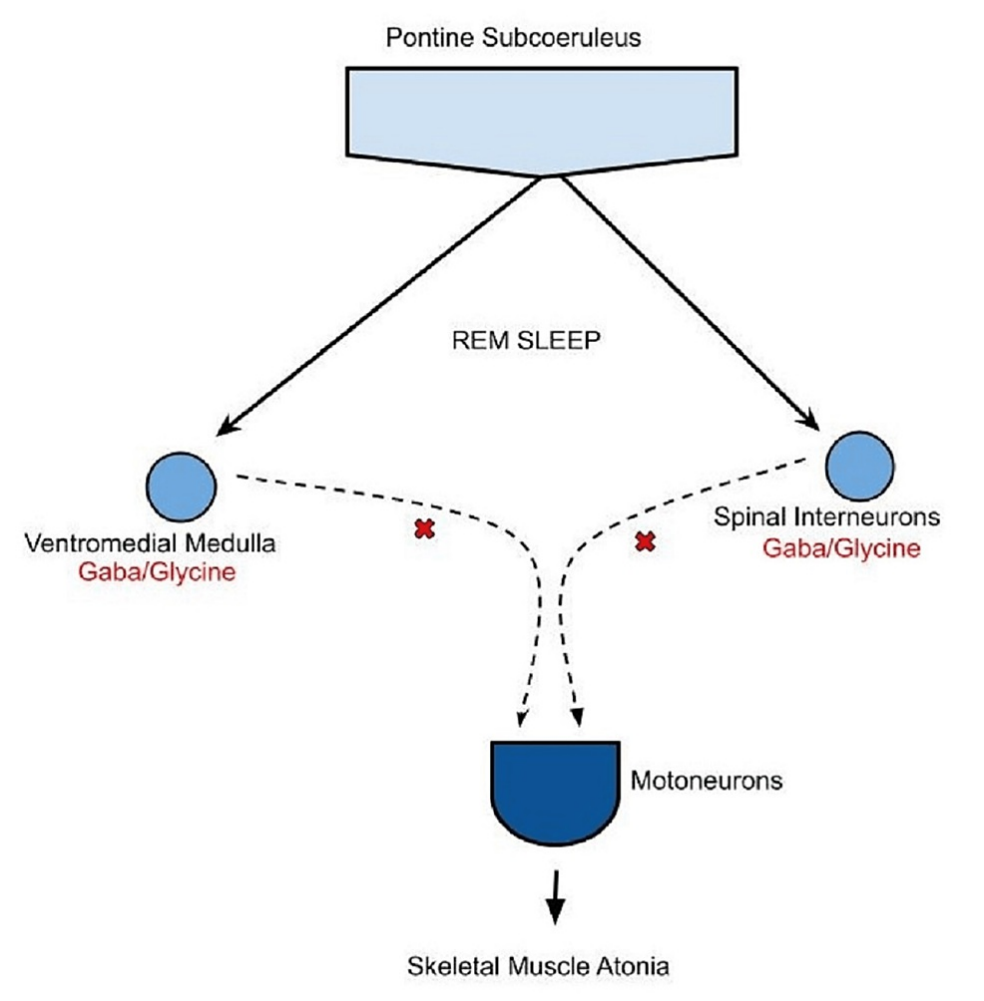
REM atonia pathway During REM sleep, the pontine subcoeruleus sends excitatory signals to GABA and glycine neurons in the ventromedial medulla and spinal interneurons, which in turn inhibit the motoneurons in the skeletal muscles leading to atonia. Image credits: Waiz Wasey, MD. REM: rapid eye movement; GABA: gamma-aminobutyric acid; x: inhibition of motoneurons by GABA and glycine.

A 50% improvement in AHI has been documented with consistent use of the intraoral stimulation device known as eXciteOSA®. What has not been documented is whether this specifically also improved REM-dependent AHI. It is well known that OSA is worse in REM sleep. This is secondary to the neurologically mediated impairment of skeletal muscles (Figure 1). The impairment is specifically due to the release of gamma-aminobutyric acid (GABA) and glycine into the skeletal motoneurons [6]. Often even though the overall severity of OSA may be mild, having a significant REM sleep AHI prompts a physician to treat it. It has not been documented what effect the neuromuscular stimulator has on REM-dependent AHI specifically.

In our patient, the overall AHI was only 10.6/hour, but the REM sleep AHI was 41.7/hour. This was why we decided to treat it with CPAP therapy initially. With the use of the intraoral stimulator, the overall AHI improved to 0.5/hour, but surprisingly, so did the REM sleep AHI. In all the sleep tests performed, the REM sleep was adequate. It is unclear whether the improvement in AHI during REM sleep was the result of increased skeletal muscle endurance or whether the stimulation somehow impeded the effects of GABA and glycine on motor neurons. Hopefully, this case study will lead to further evaluation of REM sleep AHI and intraoral neuromuscular stimulation devices to open up more treatment options for moderate to severe REM-dependent OSA.

## Conclusions

OSA is a sleep breathing disorder that is usually treated with positive airway pressure therapy. But with growing research, alternative treatment options are emerging. Daytime neuromuscular electrical training of the tongue muscles, especially the genioglossus, is one of them. It has been documented to show improvement in snoring and mild OSA with the use of a stimulation device. What has not been documented is improvement in AHI during REM sleep. AHI during REM sleep is usually worse. In our case, AHI in REM sleep was severe. Our case demonstrates the improvement in AHI overall and during REM sleep with the six-week use of eXciteOSA®. Further prospective studies are warranted to establish the role of intraoral neuromuscular stimulation therapy for moderate to severe REM-dependent OSA.
